# Optic Nerve Drusen Is Highly Prevalent Among Children With Pseudotumor Cerebri Syndrome

**DOI:** 10.3389/fneur.2021.789673

**Published:** 2021-12-13

**Authors:** Jacob Genizi, Doron Meiselles, Elisheva Arnowitz, Idan Segal, Rony Cohen, Nitza Goldenberg-Cohen

**Affiliations:** ^1^Pediatric Neurology Unit, Bnai Zion Medical Center, Haifa, Israel; ^2^Pediatric Department, Bnai Zion Medical Center, Haifa, Israel; ^3^Bruce Rappaport Faulty of Medicine, Technion, Haifa, Israel; ^4^Department of Pediatric Neurology, Schneider Children's Medical Center of Israel, Petah Tikva, Israel; ^5^Ophthalmology Department, Bnai Zion Medical Center, Haifa, Israel

**Keywords:** optic nerve drusen, pseudotumor cerebri syndrome, papilledema, pseudopapilledema, children

## Abstract

**Introduction:** The clinical presentation of pseudotumor cerebri syndrome (PTCS) usually includes headache, nausea, and vomiting with normal physical examination apart from papilledema and diplopia. However, pseudopapilledema, which can be caused by optic nerve drusen, may lead to misdiagnosis. The prevalence of optic nerve drusen in the general population is 0.5–2%. The purpose of our study was to evaluate the prevalence and risk factors of optic nerve drusen among patients with PTCS.

**Materials and Methods:** Medical records of children evaluated in the pediatric department at Bnai Zion Medical Center due to PTCS between 2008 and 2020 were assessed. Inclusion criteria were children age under 18 years with a PTCS diagnosis and ophthalmic B-mode ultrasonography (US). Exclusion criteria were secondary intracranial hypertension.

**Results:** Thirty-four children were included with a mean age 10.1 years which included 50% boys. A majority of the patients, 24 (72.4%), complained of headaches, while 15 (45.5%) complained of transient visual obscuration, and 9 (26.5%) of vomiting. Visual acuity on presentation was normal (20/20–20/30) in 23 of the children (67%), moderately diminished (20/40–20/80) in 9 (26%), and showing profound loss (20/200) in 2 (7%). Five patients (14.7%) were diagnosed with optic nerve drusen via B-mode ophthalmic ultrasonography (US). However, they still fulfilled the diagnostic criteria for PTCS, and disc swelling improved after treatment. There were no statistically significant differences between the group with optic nerve drusen and the rest of the patients.

**Conclusions:** Optic nerve drusen are common among pediatric patients with PTCS. Diagnosis of optic nerve drusen should not rule out the presence of increased intracranial pressure.

## Introduction

Pseudotumor cerebri syndrome (PTCS), or idiopathic intracranial hypertension, has been reported in children and adolescents since its publication by Dandy in 1937 (1). The fundamentals of this syndrome include the presence of increased intracranial pressure with no evidence for space-occupying lesions, hydrocephalus, or cerebrospinal fluid (CSF) pleocytosis. In 2013, Friedman et al. (2) published revised criteria for the diagnosis of PTCS, including adjustment for children. In the clinical examination, papilledema is the hallmark of increased intracranial pressure. Optic disc drusen are acellular calcified deposits buried in the surface of the optic disc, which can cause an elevated appearance of the disc (pseudopapilledema) (3–6). The prevalence of optic nerve drusen in the general population is 0.5–2% (7–11).

The link between PTCS and papilledema raises the question: does the presence of optic disc drusen rule out PTCS? Until recently, the coexistence of PTCS and optic nerve drusen was attested in only some case reports (12–17). However, two larger studies suggest that PTCS and drusen are indeed found concurrently. Birnbaum et al. (18) found drusen in 19% of the eyes they studied among 372 adults with PTCS. Gospe et al. (19), reporting on 31 pediatric patients with PTCS, identified optic nerve drusen in 48% of the eyes studied.

Recently, modern technology, such as enhanced depth imaging (EDI)-optical coherence tomography (OCT) and swept-source (SS)-OCT, and peripapillary hyper-reflective ovoid mass-like structures (PHOMS), were identified in the optic disc. They do not contain acellular calcified deposits (as opposed to drusen) and may be present in normal or elevated optic discs. It may be presumed that Gospe et al. (19) and Birnbaum et al. (18) considered PHOMS to be drusen, which might explain the high prevalence of drusen in their studies.

The aim of our study was to evaluate the prevalence and risk factors for optic nerve drusen among children and adolescents with PTCS using B mode ultrasound.

## Patients and Methods

We performed a retrospective study, reviewing medical records of children and adolescents who were diagnosed with PTCS in the pediatric department at the Bnai Zion Medical Center during the years 2008–2020. Inclusion criteria were a PTCS diagnosis based on the revised diagnostic criteria published by Friedman (2) and ophthalmic B-mode ultrasonography (US). Exclusion criteria were secondary intracranial hypertension. Optic nerve drusen were diagnosed through ophthalmic B-mode US. Collected data included demographics, patients' medical history and headache history, imaging results, lumbar puncture, and eye examination including fundoscopy. The study was approved by the Bnai Zion IRB BNZ 129-20.

## Statistical Methods

Sample characteristics were described using means, standard deviations and range for continuous variables, and frequencies and percentages for categorical variables. We used the Wilcoxon 2 sample test to test group differences for continuous data, and the chi-square test or Fisher's exact test were appropriate for categorical data. Significance was set at *p* < 0.05. Analyses were conducted using SPSS 26.0 (IBM Corporation).

## Results

Thirty-four patients fulfilled the inclusion criteria (17/34 males [50%]; mean age 10.1 years, range 1.4–17.7 years). Nineteen were prepubertal (13/19 males, 72%) and 15 were adolescents (11/15 females, 73%). Mean age on presentation was 6.3 ± 4.2 years for pre-pubertal children and 14.0 ± 2.9 years for adolescents. A statistically significant greater percentage of pre-pubertal patients were males as compared to adolescents (*p* < 0.009). Older patients were in a significantly higher weight percentile as compared to younger patients (*p* < 0.004). See Table 1.

**Table 1:** Demographic and clinical characteristics of patients with PTCS distributed by age.

	**All patients** **(*N* = 34)**	**Age 0–11** **(*N* = 19)**	**Age 12–17** **(*N* = 15)**	***X*^2^/*Z***	***P***
Age at onset	9.0 ± 8.2 (1.3–17.6)	6.3 ± 4.2 (1.3–10.6)	14.0 ± 2.9 (8.3–17.6)	–	–
**Sex**					
Male	17 (50)	13 (68.5)	4 (26.7)	6.80	0.009
Female	17 (50)	6 (31.5)	11 (73.3)		
Weight percentile (mean ± SD)	68.0 ± 29.8 (2–99)	55.9 ± 27.8 (2–99)	83.2 ± 24.1 (9–99)	2.81	0.004
Opening pressure (mean ± SD)	42.2 ± 11.8 (28–72)	40.8 ± 13.8 (28.0–72.0)	44.0 ± 9.0 (31.5–62.0)	1.50	0.14
**Complaints**					
Headache	24 (72.7)	11 (61.1)	13 (86.7)	2.69	0.11
Transient visual obscuration	15 (45.5)	7 (38.9)	8 (53.3)	0.69	0.41
Vomiting	9 (26.5)	8 (42.2)	1 (6.7)	5.41	0.02
Pulsatile tinnitus	5 (14.7)	1 (6.2)	4 (26.7)	2.31	0.13
Mean acetazolamide dose per kg	14.6 ± 4.9 (6.4–26.3)	14.7 ± 4.9 (6.4–24.0)	14.6 ± 5.0 (6.9–26.3)	0.89	0.80
Treatment time	462.0 ± 745 (11–1,775)	420.5 ± 622.5 (39–1226)	462.0 ± 1,076 (11–1,775)	0.14	0.95
Days to disappearance of symptoms	23.0 ± 155.0 (8–617)	31.5 ± 120.2 (13–588)	22.0 ± 168.0 (8–617)	0.58	0.60

A majority of the patients complained of headaches (24; 72.4%), and 45.5% (15) complained of transient visual obscuration while 26.5% (9) complained of vomiting. Vomiting was more common among young children. All other clinical symptoms were equally prevalent among young children and adolescents. All underwent CTV or MRV, ruling out space-occupying lesions or thrombosis. Lumbar puncture opening pressure was not associated with acetazolamide dose, treatment days, time to disappearance of symptoms, age, or gender. Visual acuity on presentation was normal (20/20–20/30) in 23/34 (67%), and moderately diminished (20/40–20/80) in 9/34 (26%). Two of the 34 patients (7%) had profound visual acuity loss (20/ 200).

**Figure 1:**
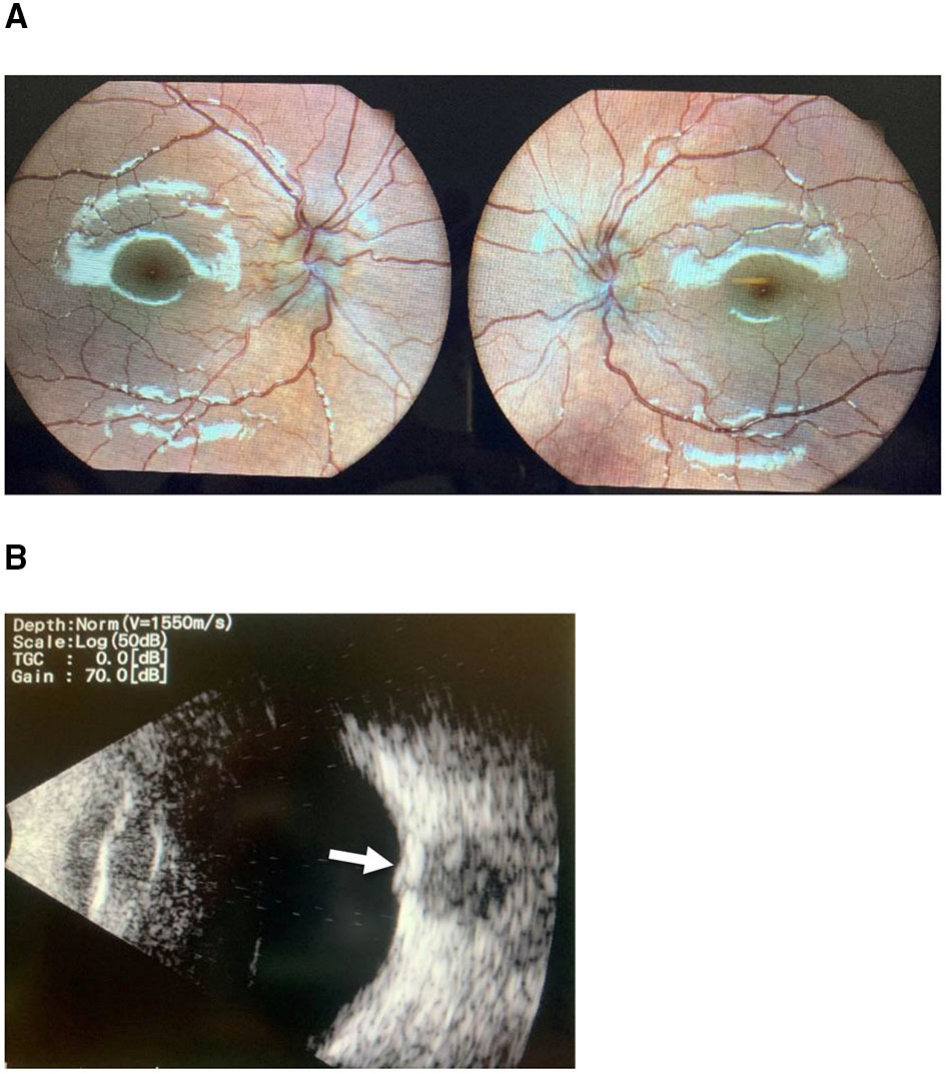
**(A)** Optic disc photography demonstrates papilledema. **(B)** B-scan ultrasonography confirming the presence of ahyper-reflective optic disc druse (arrow) in the same patient.

Five patients (14.7%) were diagnosed with optic nerve drusen. All US images were captured before the resolution of the papilledema. However, they still fulfilled the diagnostic criteria for PTCS, and disc swelling improved after treatment (Figure 1). There were no statistically significant differences in patients' age, clinical onset, complaints, gender, weight, or opening pressure between the group with optic nerve drusen and the rest of the patients. Table 2 presents the demographic and clinical characteristics of the patients by group.

**Table 2:** Demographic and clinical characteristics of patients with PTCS, with and without optic nerve drusen.

	**All patients (*N* = 34)**	**Drusen** **(*N* = 5)**	**No drusen (*N* = 29)**	***X*^2^/*Z***	***p***
Age at onset	9.0 ± 8.2 (1.3–17.6)	8.3 ± 4.3 (1.3–9.4)	9.9 ± 8.5 (2.8–17.6)	−1.19	0.25
1–11	19 (55.5)	4 (80.0)	15 (51.7)	1.38	0.24
12–17	15 (44.5)	1 (20.0)	14 (48.3)		
Gender				0.31	0.58
Male	17 (50)	2 (40.0)	15 (51.7)		
Female	17 (50)	3 (60.0)	14 (48.3)		
Weight percentile	68.0 ± 29.3 (2–99)	53.4 ± 38.8 (2–97)	70.5 ± 27.4 (9–99)	−1.02	0.32
**Complaints**					
Headache	24 (72.7)	3 (75.0)	21 (72.4)	0.01	>0.99
Transient visual obscuration	15 (45.5)	1 (25.0)	14 (48.3)	0.77	0.61
Vomiting	9 (26.5)	2 (40.0)	7 (24.1)	0.55	0.59
Pulsatile tinnitus	5 (14.7)	0 (0.0)	5 (15.63)	0.71	0.53
Other	23 (67.6)	2 (40.0)	21 (72.4)	1.99	0.16
Opening pressure (mean ± SD)	42.2 ± 11.8 (28–72)	41.2 ± 11.6 (28.0–58.0)	42.4 ± 12.1 (28.0–72.0)	0.32	0.78

Thirty-two patients (94%) were successfully managed with acetazolamide, and only two (6%) underwent CSF shunt procedures. Mean acetazolamide dose was 14.6 per kg (median 13.8; range 6.4–26.3, *N* = 32). Of these 32 patients, 19 (59.3%) were symptom-free with resolution of the papilledema at the end of follow-up (462.0 ± 745 days). Median time to symptom disappearance was 23 days (range 8–617 days).

## Discussion

In this study, we found high prevalence of optic disc drusen (14.7%) in children diagnosed with PTCS as compared to the general pediatric population (0.3–2%) (7–11). This finding might be due either to the intensive investigation of these patients secondary to headaches and other symptoms, or because drusen are associated with PTCS in children.

Optic disc drusen are acellular calcified deposits associated with disturbance in axonal flow. In children, they are typically bilateral and are more likely to be buried than in adults. Thus, they may be difficult to distinguish from true optic disc edema. On the one hand, misdiagnosing drusen as true disc edema may lead to an extensive, invasive, and unnecessary work-up for elevated intracranial pressure. On the other hand, the presence of optic disc drusen does not exclude true disc edema, and optic disc drusen may occur simultaneously with true disc edema in some children (14, 16, 20, 21). Therefore, further evaluation may be necessary even if optic disc drusen are detected (22).

In our study 14.7% of the children with PTCS were diagnosed with optic nerve drusen using B-mode ophthalmic US. This represents a very high incidence compared to the general population, where the incidence of optic nerve drusen is estimated to be 0.3–2% in adults and children (7–11).

Our findings are in concord with those of Birnbaum et al. (18), who reported that drusen were diagnosed in 19% of the eyes they studied among adults with PTCS. Gospe et al. (19) reported the presence of optic nerve drusen in as much as 48% of studied eyes among pediatric patients with PTCS. What can explain the high prevalence of optic nerve drusen among PTCS patients, given the low prevalence of drusen in the general population? Birnbaum (18) suggested that the papilledema may give rise to the formation of optic nerve drusen. Papilledema can decrease axonal transport in the optic nerve, which leads to accumulation of calcium and the formation of optic disc drusen (11).

Differences in the prevalence of optic nerve drusen in the literature might also be attributed to differences in the methods used to diagnose them. B-mode ultrasonography has generally been considered a highly sensitive imaging tool for the identification of optic nerve drusen. A newer method for drusen diagnosis is optical coherence tomography, particularly EDI-OCT and SS-OCT, which may show irregular or spheroidal hypo reflective structures in a case of drusen (11, 23, 24). Studies by Merchant et al. (24) and by Ghassibi et al. (23) using EDI-OCT suggest that up to 16% of adults in the general population may have OCT findings indicative of very small drusen that are not evident on US (25). It is also possible that ODD is far more prevalent than previously estimated.

Gospe (25) used EDI-OCT, and this method might explain the high incidence of optic nerve drusen found in their cohort. However, even in our cohort, using the classical B-mode US, a very high incidence (14.7%) of optic nerve drusen was found among patients with PTCS.

### Limitations

Our study is a small, single center, retrospective study, without a control group. A larger multicenter prospective study is planned.

## Conclusions

Optic nerve drusen are present among patients with PTCS. Hence, the diagnosis of optic nerve drusen should not rule out the presence of increased intracranial pressure.

## Data Availability Statement

The raw data supporting the conclusions of this article will be made available by the authors, without undue reservation.

## Ethics Statement

The studies involving human participants were reviewed and approved by Bnai Zion IRB BNZ 129-20. Written informed consent to participate in this study was provided by the participants' legal guardian/next of kin.

## Author Contributions

JG, DM, EA, IS, and RC: conceptualization. JG, DM, EA, IS, RC, and NG-C: methodology. JG, DM, and RC: validation. JG, DM, EA, and NG-C: investigation. JG, DM, EA, IS, RC, and NG-C: methodology and writing—review and editing and methodology. JG, DM, EA, and IS: data curation and writing—original draft preparation. All authors contributed to the article and approved the submitted version.

## Conflict of Interest

The authors declare that the research was conducted in the absence of any commercial or financial relationships that could be construed as a potential conflict of interest.

## Publisher's Note

All claims expressed in this article are solely those of the authors and do not necessarily represent those of their affiliated organizations, or those of the publisher, the editors and the reviewers. Any product that may be evaluated in this article, or claim that may be made by its manufacturer, is not guaranteed or endorsed by the publisher.
